# A proposed adaptation of the European Foundation for Quality Management Excellence Model to physical activity programmes for the elderly - development of a quality self-assessment tool using a modified Delphi process

**DOI:** 10.1186/1479-5868-8-104

**Published:** 2011-09-29

**Authors:** Ana I Marques, Leonel Santos, Pedro Soares, Rute Santos, António Oliveira-Tavares, Jorge Mota, Joana Carvalho

**Affiliations:** 1 Research Centre in Physical Activity, Health and Leisure, Faculty of Sports, University of Porto, Porto, Portugal; 2 Department of Information Systems, University of Minho, Guimarães, Portugal; 3 Department of Physical Education, Escola Secundária José Estêvão, Aveiro, Portugal; 4 Research Centre in Sports, Health Sciences and Human Development, Higher Institute of Maia, Maia, Portugal

**Keywords:** physical activity, programmes, elderly, tool, evaluation, quality, adherence

## Abstract

**Background:**

There has been a growing concern in designing physical activity (PA) programmes for elderly people, since evidence suggests that such health promotion interventions may reduce the deleterious effects of the ageing process. Complete programme evaluations are a necessary prerequisite to continuous quality improvements. Being able to refine, adapt and create tools that are suited to the realities and contexts of PA programmes for the elderly in order to support its continuous improvement is, therefore, crucial. Thus, the aim of this study was to develop a self-assessment tool for PA programmes for the elderly.

**Methods:**

A 3-round Delphi process was conducted via the Internet with 43 national experts in PA for the elderly, management and delivery of PA programmes for the elderly, sports management, quality management and gerontology, asking experts to identify the propositions that they considered relevant for inclusion in the self-assessment tool. Experts reviewed a list of proposed statements, based on the criteria and sub-criteria from the European Foundation for Quality Management Excellence Model (EFQM) and PA guidelines for older adults and rated each proposition from 1 to 8 (disagree to agree) and modified and/or added propositions. Propositions receiving either bottom or top scores of greater than 70% were considered to have achieved consensus to drop or retain, respectively.

**Results:**

In round 1, of the 196 originally-proposed statements (best practice principles), the experts modified 41, added 1 and achieved consensus on 93. In round 2, a total of 104 propositions were presented, of which experts modified 39 and achieved consensus on 53. In the last round, of 51 proposed statements, the experts achieved consensus on 19. After 3 rounds of rating, experts had not achieved consensus on 32 propositions. The resulting tool consisted of 165 statements that assess nine management areas involved in the development of PA programmes for the elderly.

**Conclusion:**

Based on experts' opinions, a self-assessment tool was found in order to access quality of PA programmes for the elderly. Information obtained with evaluations would be useful to organizations seeking to improve their services, customer satisfaction and, consequently, adherence to PA programmes, targeting the ageing population.

## Background

Physical activity (PA) programmes play a significant role in senior citizens' health, autonomy and ability to face daily tasks, being particularly important to prevent and minimize the deleterious effects of the ageing process [1,2] and to improve quality of life [1-4]. It is widely accepted that the benefits of such programmes depend on adherence to exercise, which is influenced by degree of enjoyment and satisfaction [5-10]. One of the most important factors in customer satisfaction is quality of service [11-13]. Therefore, continual improvements in PA programmes for the elderly are important to elderly satisfaction and adherence to PA.

The 3^rd ^Benchmark from the Physical Activity and Health Branch of the Centers for Disease Control and Prevention (CDC) [14] holds that complete programme evaluations are an important and desired prerequisite to continuous quality improvements. Similarly, World Health Organization (WHO) guidelines for the evaluation of health promotion emphasize the need to evaluate and propose the allocation of adequate evaluative resources [15].

Evidence shows that quality matters, is measurable, moveable and malleable [16], but also has costs [17]. However, literature also shows that the costs of not doing so are far greater [18,19]. Several studies have focused on the advantages of quality schemes [20-22]. With the aim of helping organizations improve the quality of their services, the European Foundation for Quality Management (EFQM) introduced the EFQM Excellence Model in 1991. The EFQM Excellence Model is a non-prescriptive framework that is based on nine criteria divided into 32 sub-criteria [13]. It promotes the use of management methodologies based on objective criteria that are applicable to all areas of business or services and constitutes an exercise in self-assessment. Self-assessment sheds light on areas requiring improvement, as well as on the processes and actions necessary to generate improvement.

While numerous PA programmes have been designed for the elderly in recent years - especially by the Public Local Administration - their evaluation has been scarce. In fact, few details are available on how these programmes have been developed, how they have been structured, how service delivery is conducted and how results are being achieved. The lack of a standard approach to assessing PA programmes for the elderly makes it difficult to compare the quality of both the planning and the delivery of such programmes. In this way, being able to refine, adapt and create tools that are suited to the realities and contexts of PA programmes for the elderly, and that improve these programmes, is therefore important, not only to help programmes evaluate their ability to perform public health functions, but to address local health needs and guide community health-planning efforts. Thus, the aim of this study is to describe the development of a quality self-assessment tool for PA programmes for the elderly.

## Methods

A modified Delphi process was conducted using the Internet, from October 2009 to September 2010. The Delphi technique was developed in the 1950s by scientists at the Rand Corporation as a method of making informed decisions based on expert opinion [23]. Since then, it has been used to clarify a variety of problems in different sectors [24-29]. Despite having undergone some modifications, it remains a viable approach for gathering expert opinions through a structured iterative process that builds consensus [30]. This process involves multiple interactions with participants who usually complete two or more rounds in a reasonable amount of time [31] - even when participants are in geographically-distinct locations, since rounds can be conducted by mail or email [32,33]. The results of previous iterations can be modified by participants in later iterations, as they are able to review comments and feedbacks provided by other experts in earlier rounds [31]. Furthermore, the Delphi technique offers a number of specific advantages and is particularly helpful because it avoids the barriers commonly observed in other group discussions, such as interpersonal influence, time pressure and group demands [31,34,35]. This is due to the fact that respondents are not aware of the identities of other respondents and are, therefore, freed of personal and social constraints [30]. They are also able to complete the Delphi rounds in ways that suit them best because they participate in the rounds asynchronously [36]. The Delphi technique is also advantageous because a variety of statistical analysis techniques can be used to interpret the data its generates [37].

**Figure 1:**
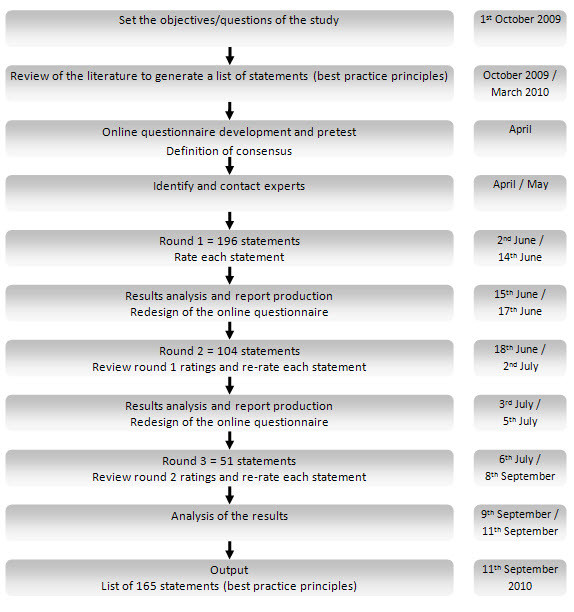
**Steps of the modified Delphi process used in the present study**.

The Delphi process was conducted in three rounds [38,39] (Figure 1). Following each step listed in the previous figure, our main question was: *Which quality practices must be included in a quality self-assessment tool for PA programmes for the elderly?*

Using criteria and sub-criteria from the EFQM Excellence Model [13] and PA guidelines for older adults [3,40] as a starting point, we reviewed the literature to identify best practice principles and generate a list of statements. Our review was undertaken using PubMed (1980-2010), B-On (1980-2010), and Google™. We searched a variety of combinations of key words related to PA programmes for the elderly, quality management and the EFQM Excellence Model, such as: 'evaluation', 'guidelines', 'recommendations', 'exercise', 'physical activity', 'programmes', 'elderly', 'old', 'review', 'framework', 'EFQM', 'assess' and 'quality'.

After identifying a list of statements, an online questionnaire was developed and tested with 5 PA programme coordinators for comments on readability and functionality. Some adjustments were made to make the affirmations included in the questionnaire clearer and more relevant to this case. We established that statements that received greater than 70% of experts' votes had achieved consensus [41-43] in both the bottom scores (i.e., reached consensus to drop) and top scores (i.e., reached consensus to include/retain). Statements that were dropped were not included in subsequent rounds of ratings. The remaining items were included in the next rounds, until a consensus was achieved to either drop or retain. At the end of three rounds, the statements on which experts had not reached consensus were also not included in the output list.

The fourth phase of the process involved nominating experts to participate in the Delphi rounds. National experts in research on PA for the elderly, PA programmes for elderly management and delivery, sports management, quality management and gerontology were identified. Our decisions were based on expertise or/and breadth of scientific work [44]. The DeGóis Curricula Platform^1 ^assisted us in this process. A list of 63 potential participants was generated, along with key contacts for each. This group included 34 PhD scientists and academics (11 in PA for the elderly, 4 in sports management, 18 in quality management and 1 in gerontology), 3 non-PhD academics (1 in PA for the elderly and 2 in sports management) and 26 senior technicians (22 in PA programmes for elderly management and delivery, 3 in quality management and 1 in gerontology). Previous information containing details about the EFQM Excellence Model, the Delphi process and the purpose of our study was provided. Of those invited to participate, 5 did not respond and 3 declined, due to lack of time (all PhD scientists and academics in quality management). Thus, 55 experts (30 females and 25 males) responded to our initial invitation and agreed to participate. Those who accepted our invitation were informed that they were required to respond to three online rounds of ratings.

The rounds were performed using Survey Monkey, a web-based survey and data collection system. In every round, participants were asked to rate their level of agreement with each proposition, from 1 to 8 ('strongly disagree' to 'strongly agree'), suggest modifications to proposed definitions and/or add propositions that would be useful in a quality self-assessment tool for PA programmes for the elderly. The 8-point Likert scale was selected to bring out more variability in responses [45]. After each round, the frequency and mean of the panel's ratings and the percentage of scores ≥ 7 were calculated. Based on this data, a new questionnaire was designed and placed online for the next round. We asked participants to review all the information sent and re-rate each statement.

After round 3, we gathered all our data and developed a list of statements that did and did not reach consensus.

## Results

Eight of the 63 invited experts, did not respond or declined. Of the 55 who agreed to participate in this process, 43 responded to round 1 and were invited to participate in the subsequent rounds. This group included 25 females and 18 males and was comprised of 20 PhD scientists and academics (9 in PA for the elderly, 2 in sports management, 8 in quality management and 1 in gerontology), 2 non-PhD academics (1 in PA for the elderly and 1 in sports management) and 21 senior technicians (17 in PA programmes for elderly management and delivery, 3 in quality management and 1 in gerontology). The 12 experts who did not respond to round 1 were not involved in subsequent rounds.

The results of the three rounds (total number of statements, statements approved by consensus, statements without consensus, statements modified by experts and new statements proposed by experts) for the nine criteria are presented in Table 1.

**Table 1:** Results of the three rounds by criterion

		LEADERSHIP	POLICY & STRATEGY	PEOPLE	PARTNERSHIP & RESOURCES	PROCESSES	CUSTOMER RESULTS	PEOPLE RESULTS	SOCIETY RESULTS	KEY PERFORMANCE RESULTS	TOTALS
1^st ^ROUND	With consensus	13	12	18	15	27	3	3	1	1	93
	Without consensus	9	7	12	11	13	2	5	2	1	62
	To modify	14	9	7	0	7	1	1	0	2	41
	To add	0	0	1	0	0	0	0	0	0	1
	**Total**	**36**	**28**	**37**	**26**	**47**	**6**	**9**	**3**	**4**	**196**

2^nd ^ROUND	With consensus	14	5	14	2	12	2	2	0	2	53
	Without consensus	8	8	4	6	5	1	4	2	1	39
	To modify	1	3	2	3	3	0	0	0	0	12
	To add	0	0	0	0	0	0	0	0	0	0
	**Total**	**23**	**16**	**20**	**11**	**20**	**3**	**6**	**2**	**3**	**104**

3^rd ^ROUND	With consensus	2	5	2	4	4	1	0	1	0	19
	
	Without consensus	7	6	4	5	4	0	4	1	1	32
	
	**Total**	**9**	**11**	**6**	**9**	**8**	**1**	**4**	**2**	**1**	**51**

In round 1, of the 196 originally-proposed statements (best practice principles), the experts modified 41, added 1 and achieved consensus on 93, which were retained for inclusion in the self-assessment tool. Of the 41 suggested modifications, 14 were related to Leadership (38,39%), 9 to Policy & strategy (32,14%), 7 to People (18,92%), 7 to Processes (14,89%), 1 to Customer results and People results (16,67% and 11,11 respectively) and 2 to Key performance results (50%). Some modifications consisted of minor changes to words or sentence structures, while others were about content (e.g., change "Higher education qualification, with specialization in physical activity and aging, is required for instructors'/teachers' programmes" to "Higher education qualification, with specialization in physical activity and aging, or relevant experience in this field, is required for instructors'/teachers' programmes". The addition was related to the People criterion. Generally, experts made the greatest number of suggestions to Leadership and the fewest (0 in this case) to Partnership & resources and Society results. The best practice principles that were retained were mostly in Partnership & resources (15 out of 26, i.e. 57,69%), Processes (27 out of 47, i.e. 57,45%) and Customer results (3 out of 6, i.e. 50%). The criterion on which least consensus was reached was Key performance results (1 out of 4, i.e. 25%). No proposition was dropped in round 1, i.e. none received greater than 70% of the experts' votes in both the bottom scores.

Based on the results of round 1, 104 propositions were presented in round 2. At this stage, experts modified 39 and achieved consensus on 53 propositions. Most of the suggestions were made on Policy & strategy, Partnership & resources and Processes, with none suggestions to Results' criteria. The best practice principles that were retained were mostly in People (14 out of 20, i.e. 70%), Leadership (14 out of 23, i.e. 60,87%) and Processes (12 out of 20, i.e. 60%). The criterion on which there was least consensus was Society results, on which there was no agreement. Once more, no proposition was dropped. Forty one of the 43 experts responded to round 2.

In the last round, of the 51 statements proposed, the experts achieved consensus on 19, mostly in Policy & strategy (5 out of 11, i.e. 45,45%), Processes (4 out of 8, i.e. 50%) and Partnership & resources (4 out of 9, i.e. 44,44%). After 3 rounds of rating, they had not achieved consensus on 32 propositions. Most of these statements were concerned with Leadership (7, i.e. 21,88%), Policy & strategy (6, i.e. 18,75%) and Partnership & resources (5, i.e. 15,63%). One expert who had not responded to round 2 was willing to participate in round 3; thus, 42 of the 43 experts responded to round 3.

Additional file 1 presents the resulting tool - named Q-STEPS (Quality **S**elf-assessment **T**ool for **E**xercise **P**rogrammes for **S**eniors) - which consists of 165 statements that assess nine areas involved in the development of PA programmes for the elderly. Five criteria assess *Enablers *(Leadership, Policy & strategy, People, Partnership & resources, and Processes) and four criteria assess the *Results *(Customer results, People results, Society results, and Key performance results).

## Discussion

The main goal of this study was to describe the development of a quality self-assessment tool for PA programmes for the elderly. To the best of our knowledge, no previous studies have sought expert opinions on PA for the elderly, PA programmes for elderly management and delivery, sports management, quality management and gerontology, with the aim of identifying practices that must be observed when assessing the quality of such programmes.

Although there are recommendations and guidelines for promoting the physical activity of older people [3,40] and recommendations about the need to evaluate these interventions [14,46], the literature is scarce [47], if not absent, on how to integrate these recommendations into PA programmes. No framework or tool has yet been developed to identify or influence the enablers and outcomes of PA programmes for the elderly.

The 43 national experts who participated in the Delphi process were quite engaged throughout, as evidenced by the number of their suggestions (one addition and 53 modifications) and the greater than 97% response rate to all three rounds of ratings. Most of their suggestions pertained to *Leadership*, while they made no suggestions on *Society results*. We presume that these results are related to the fact that many experts are programme leaders and thus, are more aware of practices that pertain to Leadership. Also, experts may have been aware of the fact that Leadership is understood by some authors [48-50] as the key to driving quality improvement. Our data indicate a high degree of consensus on the retention of all propositions concerning the development of vision and mission and the enhancement of a culture of communication by programme coordinators. These are considered fundamental to quality management [51-53], since the physical presence of leaders - their visibility and concern for quality improvement - are associated with transformational leadership [54], i.e. leadership that creates valuable and positive change in its followers. Of the seven statements on Leadership on which experts did not achieve consensus, five belong to the sub-criteria that concern the interaction of programme coordinators with politicians, customers, partners and representatives of society. While our study revealed that most of the statements concerning interaction with customers, partners and representatives of society achieved consensus, propositions concerning relationships with politicians or political affairs did not achieve consensus. This may be related to popular negative perceptions of the political class [55]. Examples of statements that touched on the relationship between leadership and politics include "The coordinator manages relations with politicians and other stakeholders to ensure shared responsibility" and "The coordinator interacts regularly and proactively with policy makers from relevant executive areas (e.g. Alderman of Sport)". The British Heart Foundation (BHF) has stated that participants or other stakeholders must be actively involved in all aspects of programme development, including planning, promotion and evaluation [40]. The ACSM also recognizes that PA leaders should work closely with individuals to design PA regimens that reflect personal preferences and capabilities [56].

Leaders unanimously agreed to retain statements about the importance of leaders identifying and championing organizational change. Fostering change is increasingly seen as part of a leader's role [57], and the EFQM Fundamental Concepts upon which the Model is based [58] include standard recommendations such as planning change, communicating reasons for it, enabling people to manage change and reviewing the effectiveness of change.

Experts also suggested modifications to about 30% of the original propositions on *Policy & strategy*. A high degree of consensus was achieved on the retention of all propositions concerning the development, review and updating of policy and strategy.

The statement that received the greatest degree of consensus was related to the development of annual reports. Data from such reports helps improve the annual planning cycles of PA programmes. These procedures are in agreement with those found in other studies [59,60] or with different documents, such as those that outline the planning and evaluation of PA programmes [61,62] and health promotion programmes [63].

Throughout the Delphi process, it was suggested that the proposition "The programme involves a multidisciplinary team of professionals" be added to the *People *criterion. In fact, the teams that run PA programmes for seniors should include not only exercise and sports professionals, but general practitioners, practice nurses and care and residential managers [40]. Of the propositions on the planning, management and improvement of human resources that the experts agreed to retain, the one on which there was greatest consensus was "Emphasis is placed on recruiting employees whose profile matches the needs of the programme". The Physical Activity and Health Branch (PAHB) of the CDC has established that PA programmes should be run by highly-skilled PA practitioners [14]. The *Cross-National Expert Survey Report on Physical Activity Programmes and Physical Activity Promotion Strategies for Older People *[64] also notes the importance of recruiting teachers who are highly qualified and reinforces the importance of continuous professional development.

During the first round, a high level of consensus was immediately reached on propositions related to the management of finances and maintenance of facilities, equipment and materials (*Partnership & resources *criterion). The management of financial resources is key to consolidating programmes' financial structure and ensuring that programmes can fulfil their missions in the present and the future, as well as periodically provide maintenance plans for equipment and buildings [65,66]. Experts did not achieve consensus on half the propositions concerning "external partnerships", although the development and sustainment of community partnerships is the first public health benchmark for PA programmes established by the PAHB [14]. Particularly with regards to PA programmes for the elderly, some organizations have reinforced the importance and strength of these partnerships, which provide additional resources in the form of funding, facilities and equipment, as well as access to wide-ranging abilities and knowledge [40,67]. Indeed, one of the propositions that did not reach consensus was the one that pointed the participation in networks in order to exchange knowledge and to improve relationships. However, of the propositions on which experts did not achieve consensus, most were similar to other statements that were retained. Examples include: "Appropriate partnership agreements are established, defining roles, responsibilities and expected outcomes" and "Regular and formal communication procedures are established with partners".

Consensus was not reached on only four of the 47 statements about *Processes*. Once more, most were similar to other statements that were retained. For example, "Market research is used to determine the needs and expectations of future customers" -- a proposition that only received 64,29% of votes equal to or greater than 7 -- is comparable to "Surveys and other ways of obtaining feedback are used to determine the needs and expectations of current and future customers", a retained proposition. Physical activity leaders should work closely with individuals to design PA regimens that reflect personal preferences and capabilities [56]. The BHF recommends that participants should be involved in this process [40]. Moreover, tailoring exercise programmes to the needs and interests of participants has been associated with higher programme attendance [68,69].

Concerning the four Results' criteria, the highest level of consensus was achieved on *Customer results*, in which all propositions were accepted. Indeed, organizations must measure and achieve customer results [13]. Similarly, both the processes by which PA interventions are conducted and the outcomes of such interventions should be evaluated [47]. The experts achieved a high degree of consensus on all propositions related to client assessment, i.e. customer satisfaction, customer loyalty, communication, complaints handling and management and outcomes (physical fitness evaluations and psychological/mental evaluations). By contrast, they displayed relatively little consensus on the criterion *People results *(4 out of 9). In fact, the experts were unable to reach consensus on whether or not to retain propositions related to employee involvement, motivation, initiative and loyalty. However, it should be emphasised that similar statements were retained. Examples include: "The programme has measures of perception and/or performance indicators regarding employees' performance" and "The programme has measures of perception and/or performance indicators regarding employees' involvement in teamwork". In actuality, to achieve excellence, organisations must also focus on People results [13], since employee involvement is one of the most important drivers of continuous improvement [58]. Furthermore, without satisfied and motivated employees, it is impossible to create satisfied and loyal customers [70].

The tool that resulted from this process provides a framework tailored to evaluating PA programmes for the elderly, applicable to a variety of settings, namely community-based programmes and/or those developed by the Public Local Administration. The information obtained through such evaluations would be useful for organizations seeking to improve their services. It would help them guide interventions toward excellence, in order to improve customer satisfaction and adherence to PA programmes targeting the ageing population.

### Strengths and Limitations

To the best of our knowledge, this is the first study to gather expert opinions with the aim of identifying practices that must be observed when assessing the quality of PA programmes for the elderly. Because of the heterogeneity of their interests, panel members were able to cover a broad range of topics. In addition, they were able to submit comments on each sub-criterion in every round, enabling us to use their expertise to develop or modify new statements. This also guaranteed that the process did not neglect to include any pertinent issues in subsequent rounds of rating.

However, this study has certain limitations. Our results should not be interpreted as representing the views of all experts in the field of quality management, physical activity for older adults or gerontology, due to the process used to collect the sample. It is also important to note that the tool suggested by our consensus process may not be applicable to certain PA programmes, including those for special population subgroups, such as: the most elderly, the frail, older adults with chronic illnesses or varying degrees of medical co-morbidity. Likewise, our consensus-informed quality practices do not reflect possible differences in PA programmes that were developed in institutional elderly care settings. Additional research is necessary to provide the feasibility analysis of this assessment and to adapt and replicate our tool to other circumstances.

## Conclusion

Our Delphi process identified 165 quality practices that 43 experts consider essential to assessments of the quality of PA programmes for the elderly. The Q-STEPS (Quality **S**elf-assessment **T**ool for **E**xercise **P**rogrammes for **S**eniors) tool assesses nine areas involved in the development of PA programmes for the elderly: five criteria assess Enablers (Leadership, Policy & strategy, People, Partnership & resources, and Processes) and four criteria assess the Results (Customer results, People results, Society results, and Key performance results).

## Competing interests

The authors declare that they have no competing interests.

## Authors' contributions

AIM, LS, PS, and JM conceptualized and contributed to the design of this study. AIM participated in the acquisition and analysis of data and participated in drafting and editing the manuscript. AOT and RS managed the data collection and analysis. JC participated in the coordination of the study and supervised the drafting and editing of manuscript. All authors reviewed and revised drafts of the manuscript. All authors read and approved the final manuscript.

## Ethics approval

The study was approved by the Scientific Council and Ethics Committee of the Faculty of Sport - University of Porto.

## Endnotes

^1 ^It is an instrument for gathering, supplying and analyze the intellectual and scientific production of the Portuguese researchers.

## Supplementary Material

Additional file 1 **Q-STEPS (Quality Self-assessment Tool for Exercise Programmes for Seniors)**. the file presents the resulting tool - named Q-STEPS - which consists of 165 statements that assess nine areas involved in the development of PA programmes for the elderly. Click here for file
