# Supplementary material for: A proposed adaptation of the European Foundation for Quality Management Excellence Model to physical activity programmes for the elderly - development of a quality self-assessment tool using a modified Delphi process
Source: Int J Behav Nutr Phys Act. 2011 Sep 29;8:104. doi: 10.1186/1479-5868-8-104 (PMC3278362; doi:10.1186/1479-5868-8-104)
Supplement: Additional file 1 — Q-STEPS (Quality Self-assessment Tool for Exercise Programmes for Seniors). the file presents the resulting tool - named Q-STEPS - which consists of 165 statements that assess nine areas involved in the development of PA programmes for the elderly. [file 1479-5868-8-104-S1.DOC]

LEADERSHIP

| **1a. The programme coordinator develops the mission, vision, values, and ethics and is a role model for a culture of Excellence** |
| --- |
| 1. The coordinator identifies the programme's mission |
| 2. The coordinator identifies the programme’s vision |
| 3. The coordinator identifies a set of values for programme operationalization |
| 4. The coordinator acts as a model in developing a culture of Excellence |
| 5. The coordinator encourages empowerment |
| 6. The Ccoordinator promotes and encourages innovation and creativity in the team |
| 7. The coordinator encourages collaboration between team members |
| 8. The coordinator participates and gives support to improvement processes |
| 9. The coordinator collaborates in the training of team members |
| 10. The coordinator is involved in creating an identity for the programme, ensuring that all team members have a sense of belonging to it |

| **1b. The programme coordinator is personally involved in ensuring the organization’s management system is developed, implemented, and continuously improved** |
| --- |
| 1. The coordinator ensures the implementation of a process to measure, review, and improve programme results |
| 2. The coordinator ensures the implementation of best practices in programme management |
| 3. The coordinator is committed and involved in continuous improvement of the programme, supporting the necessary mechanisms |

| **1c. The programme coordinator interacts with the politicians, customers, partners, and representatives of society** |
| --- |
| 1.The coordinator understands and responds appropriately to the needs and expectations of stakeholders |
| 2.The coordinator ensures that the programme's strategy is aligned with public policy |
| 3.The coordinator is committed to the satisfaction of both current and future customers |
| 4.The coordinator establishes partnerships and participates in networks related to the programme |
| 5.The coordinator acknowledges the contribution of stakeholders |
| 6.The coordinator participates in conferences and other events, looking for public disclosure, reputation, and recognition of the programme and its services |

| **1d. The programme coordinator reinforces a culture of excellence with the organization’s people** |
| --- |
| 1. The coordinator communicates personally to team members the mission, vision, values, policy and strategy, plans, objectives, and targets to achieve |
| 2. The coordinator encourages a culture of communication among all team members |
| 3. The coordinator acts to enable the integration and mobilization of team members |
| 4. The coordinator encourages and facilitates the participation of team members in improvement activities (e.g. suggestion schemes) |
| 5. The coordinator recognizes and values both the individual and team work |

| **1e. The programme coordinator identifies and sponsors the change** |
| --- |
| 1. The coordinator acquires and continually updates the knowledge, identifying the internal and external factors of change |
| 2. The coordinator identifies and sets priorities for the necessary changes |
| 3. The coordinator involves team members in the definition of plans for change |
| 4. The coordinator communicates the plans for change to all stakeholders |
| 5.The coordinator ensures the necessary resources to developing and implementing plans for change |

POLICY AND STRATEGY

| **2a. Policy and strategy are based on the present needs and future expectations of stakeholders** |
| --- |
| 1. The coordinator identifies all relevant stakeholders |
| 2. The strategy, plans, and objectives of the programme are developed in accordance with international, national, regional, and local physical activity and health policies |
| 3. The strategy, plans, and objectives of the programme are developed through consultation with relevant external partners |
| 4. The needs and customer expectations are taken into account when setting programme objectives |

| **2b. Policy and strategy are based on information from performance measurement, research, learning, and external related activities** |
| --- |
| 1. Procedures are in place to capture external information (about customers, partners, society, etc.) |
| 2. The results of the programme’s performance indicators are analyzed |
| 3. The strategic objectives and plans take into account national, regional, and local policies and strategies |
| 4. The strategic objectives and plans take into account the past performance of the programme |
| 5. Procedures are implemented to allow comparison with other programmes (Benchmarking) |
| 6. Data about organizational learning activities are analyzed |

| **2c. Policy and strategy are planned, reviewed, and updated** |
| --- |
| 1. Systematic procedures are in place to plan, evaluate, and monitor the effectiveness of the programme |
| 2. The stakeholders are involved in the planning and programme evaluation |
| 3. The qualitative and quantitative methods are used for collecting data for the evaluation of programme effectiveness |
| 4. The strategic objectives and plans are reviewed regularly to identify their relevance and effectiveness |
| 5. There is an annual plan of activities |
| 6. There is a programme activity report at the end of each year |
| 7. The continuous improvement processes are based on systematic evaluation of programme effectiveness |
| 8. A annual programme quality self-assessment is implemented |

| **2d. Policy and Strategy are communicated and deployed through a framework of key and support processes** |
| --- |
| 1. The policy and strategy outlined for the programme are effectively communicated to all stakeholders |
| 2. The policy and strategy outlined for the programme are operationalized through a set of interrelated key processes |
| 3.The key processes and procedures to support the programme and their interrelationships are identified and defined |
| 4.Those responsible for planning, coordination, and performance improvement of each process are designated |

PEOPLE

| **3a. Human resources are planned, managed, and improved** |
| --- |
| 1. The current and future human resources deficiencies are regularly reviewed, taking into account the needs and expectations of stakeholders |
| 2.There is a clear policy containing objective criteria in the areas of recruitment, promotion, remuneration, evaluation, and delegation |
| 3. Equity and equal opportunities are ensured to all employees |
| 4. Formal processes are used to ﬁnd out employees’ opinions |
| 5. Emphasis is placed on recruiting employees whose profile matches the needs of the programme |
| 6. Higher education qualification, with specialization in physical activity and aging, or relevant experience in this field, is required for instructors’/teachers’ programmes |
| 7. The programme involves a multidisciplinary team of professionals |

| **3b. People’s knowledge and competences are identiﬁed, developed, and sustained** |
| --- |
| 1. The actual competencies of employees at an individual and organizational level are identified, in terms of knowledge, skills, and attitudes |
| 2. Employees have the necessary skills, knowledge, and experience necessary to perform their tasks |
| 3. The competencies and responsibilities of employees are documented, reviewed, and updated regularly |
| 4. Development plans and training are developed and agreed for all employees |
| 5. Training means are provided for employees |
| 6.The employees continuously update their skills in their specific area of knowledge |
| 7. Skills for teamwork are developed |
| 8.The new employees are supported and monitored |
| 9. Employees’ performance is evaluated annually |

| **3c. People are involved and empowered** |
| --- |
| 1. Appropriate mechanisms are developed to allow input from employees |
| 2.The employees have the opportunity to suggest and implement solutions to solve problems |
| 3.The employees are involved in developing plans, strategies, and objectives of the programme |
| 4.The employees are involved in identifying and implementing improvement actions |
| 5. Employees’ autonomy is encouraged |
| 6. Employees' creativity is encouraged |
| 7.The teamwork is encouraged |

| **3d. People and the organization have a dialogue** |
| --- |
| 1. Formal communication procedures are established among employees |
| 2. Employees have access to information about programme performance |
| 3. Employees have access to information about quality initiatives results |
| 4. There are formal communication channels to provide customer information to the employees |
| 5. Employees maintain a fluid communication, going beyond the formal structure of the organization |
| 6.The internal communication is open and transparent |
| 7. Employees voluntarily pass on useful information between one another |
| 8. Best practices and knowledge is shared |

| **3e. People are rewarded, recognized, and taken into consideration** |
| --- |
| 1. There is an employee motivation policy, with options and concrete actions |
| 2. The merits of the employees are recognized to sustain their involvement and accountability |
| 3.There is a systematic procedure to measure employee satisfaction |

PARTNERSHIPS AND RESOURCES

| **4a. External partnerships are managed** |
| --- |
| 1. Appropriate partnership agreements are established, defining roles, responsibilities, and expected outcomes |
| 2. Regular and formal communication procedures are established with partners |
| 3. Regular monitoring and evaluation are conducted concerning the partnerships’ processes and outcomes |

| **4b. Finances are managed** |
| --- |
| 1. Funding sources for the programme are identified |
| 2.The financial processes are designed and managed to ensure efficiency and effectiveness |
| 3.The financial mechanisms are in place to ensure effective use of resources, avoiding unnecessary costs |
| 4.The cost-benefit of each action is assessed |
| 5.The financial resources are allocated to different processes based on predefined criteria |

| **4c. Facilities, equipment, and materials have a maintenance plan** |
| --- |
| 1. There are procedures for the proper management and maintenance of facilities, equipment, and materials |
| 2. The maintenance plans are documented and periodically reviewed |
| 3. The facilities are properly managed for the benefit of the programme (e.g., decentralization of facilities / services) |
| 4. The equipment and materials are properly managed and updated |
| 5. The facilities, equipment and materials are considered sufficient for the proper development of the programme’s processes and activities |
| 6. Adequate accessibilities to buildings / facilities are ensured, taking into account the needs and expectations of employees and customers (e.g. access for the disabled and people with reduced mobility, parking lots, sidewalks, and bike lanes or public transport accessibility) |

| **4d. Technology is managed** |
| --- |
| 1. When appropriate, technological innovations are implemented |
| 2.The team members use the information and communication technologies to support decisions |
| 3.The Internet is used to support communication with different stakeholders |

| **4e. The information and knowledge are managed** |
| --- |
| 1. Procedures are implemented to ensure adequate access to information and knowledge, respecting ethical issues |
| 2. Regular records of information and knowledge are conducted |
| 3. There is a systematic updating of technical and scientific knowledge of the intervention |
| 4. Appropriate access to information and knowledge is provided to relevant stakeholders |

PROCESSES

| **5a. Processes are systematically designed and managed** |
| --- |
| 1.The processes are described and documented regularly |
| 2. There are documents and formal systems which manage and control the processes |
| 3.The processes, procedures, and working methods are clearly defined and effectively implemented |
| 4.The responsibility for coordinating, monitoring, and reviewing processes are clearly defined |
| 5. Measurement processes, evaluation, and quality control are implemented |
| 6. Performance indicators are established |
| 7.The processes are periodically reviewed |

| **5b. The processes are improved through innovation in order to fully satisfy and generate increasing value for customers and other stakeholders** |
| --- |
| 1. Opportunities for improvement and other changes are identified |
| 2. The information from the learning activities is used for process improvement |
| 3. The performance indicator results and measures of perception are used to improve processes |
| 4. The procedures are improved according to the efficiency, effectiveness, and outcomes |
| 5. Necessary resources are allocated to process innovation |
| 6. Changes in procedures are communicated to all stakeholders |

| **5c. Services are planned and developed based on customers’ needs and expectations** |
| --- |
| 1. Surveys and other ways of obtaining feedback are used to determine the needs and expectations of current and future customers |
| 2. New products and services of the programme are designed and developed to meet the needs and expectations of customers |
| 3. Adequate and accurate information is ensured to meet the needs of customers |

| **5d1. The services are produced, delivered, and serviced (Health and Safety)** |
| --- |
| 1. The working methods are based on legislation and / or scientific recommendations to ensure the safety of customers |
| 2. The programme activities developed all of the following components: aerobic fitness, strength, balance, and flexibility |
| 3. The initial screening for risk assessment is intended to ensure the safe participation of customers (eg questionnaires PAR-Q, RAPA) |
| 4. Each training session is divided into phases: general activation (including stretching), main, and return to calm |
| 5.The principle of progression concerning complexity and intensity is observed |
| 6. Good environmental conditions are guaranteed in places for the sessions, such as temperature, humidity, lighting, acoustics, and access to potable water |
| 7. The program develops strategies for behavior change which may include: social support, self-efficacy, health contract, positive reinforcement, etc… |
| 8.The emergency protocols are documented, periodically reviewed, and tested |
| 9.The team members regularly review health and safety data and act accordingly |

| **5d2. The services are produced, delivered, and serviced (Administration and Marketing)** |
| --- |
| 1. Simplified administrative procedures are implemented |
| 2. Communication between the organization and the customer is encouraged through various means, including e-mail |
| 3. Means of access to the programme are promoted, such as flexible schedules and formative/informative documents in different formats and methods |
| 4. There are several ways for customers to access the programme (eg, decentralized municipal service) |
| 5. Tariffs applied in the programme ensures the principles of fairness, justice, and participation |
| 6. There are different ways to publicize the programme |
| 7. Appropriate and realistic images are used in promotional materials |
| 8. National, regional, or local publicity campaigns are used in conjunction with the programme’s marketing, when appropriate |
| 9. A web page with all the information and publicity about the programme is developed, promoted, and maintained |
| 10. Actions and events are promoted effectively, achieving visibility |

| **5e. Customer relationships are managed and enhanced** |
| --- |
| 1. Customer satisfaction is a priority |
| 2. A proactive involvement with customers is developed in order to discuss and respond to their needs and expectations |
| 3. Team members manage relationships with customers in a positive manner to provide a professional and friendly service |
| 4. Team members have access to relevant and updated information about the programme to answer customers' questions |
| 5. Mechanisms are developed to respond to questions and procedures |
| 6. Team members deal with customers’ feedback promptly |
| 7. Standardized systems are in place to deal with customer complaints |
| 8. Standardized systems are in place to deal with customer suggestions |

CUSTOMER RESULTS

| **6a. Customer Results** |
| --- |
| 1. The programme has measures of perception and / or performance indicators of customer satisfaction |
| 2. The programme has measures of perception and / or performance indicators of customer loyalty |
| 3. The programme has measures of perception and / or performance indicators about communication with the customer |
| 4. The programme has measures of perception and / or performance indicators of the complaint resolution process |
| 5. The programme has performance indicators of clients’ physical fitness |
| 6. The programme has measures of perceptions of clients’ psychological health and / or welfare |

PEOPLE RESULTS

| **7a. People Results** |
| --- |
| 1. The programme has measures of perception and/or performance indicators regarding employees’ training |
| 2. The programme has performance indicators of employees’ performance (e.g., appraisals) |
| 3. The programme has measures of perception and / or performance indicators of employee satisfaction |
| 4. The programme has performance indicators of employee absenteeism |
| 5. The programme has measures of perception and/or performance indicators regarding employees’ involvement in teamwork |

SOCIETY RESULTS

| **8a. Society Results** |
| --- |
| 1. The programme has measures of perception and / or performance indicators of their involvement in the community (e.g. social support, participation, and training of community members, etc.) |
| 2. The programme has measures of perception and / or performance indicators of their social responsibility (e.g. positive discrimination charges for needy elderly) |

KEY PERFORMANCE RESULTS

| **9a. Financial Results** |
| --- |
| 1. The programme has performance indicators on its financial performance |

| **9b. External results** |
| --- |
| 1. The programme has measures of perception and / or performance indicators of the quality of service provided |

| **9c. Results on processes** |
| --- |
| 1.The programme has measures of perception and / or performance indicators of efficiency and effectiveness of its processes |
